# MicroRNA-150 Modulates Adipogenic Differentiation of Adipose-Derived Stem Cells by Targeting Notch3

**DOI:** 10.1155/2019/2743047

**Published:** 2019-10-30

**Authors:** Xiang Li, Yu Zhao, Xiuquan Li, Qiao Wang, Qiang Ao, Xiaohong Wang, Xiaohong Tian, Hao Tong, Deyu Kong, Shijie Chang, Shuling Bai, Jun Fan

**Affiliations:** ^1^Department of Cell Biology, Key Laboratory of Cell Biology, Ministry of Public Health, and Key Laboratory of Medical Cell Biology, Ministry of Education, China Medical University, Shenyang, Liaoning 110122, China; ^2^Department of Tissue Engineering, School of Fundamental Science, China Medical University, Shenyang, Liaoning 110122, China; ^3^Department of Plastic Surgery, Shengjing Hospital, Affiliated Hospital of China Medical University, Shenyang, Liaoning 110004, China; ^4^Department of Biomedical Engineering, School of Fundamental Science, China Medical University, Shenyang, Liaoning 110122, China

## Abstract

MicroRNAs (miRNAs) influence stem cell functions, including mobilization, proliferation, and differentiation. miR-150 is abundantly expressed in monocytes. Knockdown of miR-150 promotes bone marrow stem cell migration. The role of miR-150 in adipose-derived stem cells (ADSCs) is unclear. In this study, the effects of miR-150 on adipogenic differentiation and proliferation of ADSCs were investigated. ADSCs were isolated from the inguinal adipose tissue of wild-type (WT) and miR-150 knockout (KO) mice and were induced for adipogenic differentiation. The miR-150 level was detected by real-time PCR. ADSCs were transfected by miR-150 or small-interfering RNA (siRNA) of Notch3. MTT assay and colony formation assay were performed in miR-150 knockdown and control ADSCs. Real-time PCR showed that miR-150 was expressed in ADSCs. miR-150 knockdown significantly decreased the capacity of adipogenic differentiation of ADSCs, as compared with their counterparts from WT mice. It is intriguing that the overexpression of miR-150 significantly increased C/EBP*α* and PPAR-*γ* expression and lipid formation in ADSCs with adipogenic induction. Overexpression of miR-150 significantly decreased Notch3 expression in ADSCs compared with the control groups. Furthermore, Notch3 inhibition promoted the adipogenic differentiation in ADSCs. miR-150 also suppressed proliferation potential and the expression of Nanog in ADSCs. In summary, this study demonstrates, for the first time, that miR-150 promotes adipogenic differentiation and inhibits proliferation of ADSCs. miR-150 regulates adipogenic differentiation of ADSCs, likely mediated by the downregulation of Notch3.

## 1. Introduction

ADSCs are a population of mesenchymal stem cells found in adipose tissue and are readily accessible from subcutaneous liposuction. ADSCs can self-renew and can differentiate into a number of cell types, which make them a viable option in regenerative medicine. The investigations show that ADSCs are a promising cell source for adipose tissue engineering as they can be differentiated into adipocytes [1]. Understanding the mechanism of adipogenic differentiation in ADSCs may provide means to develop strategies for the therapeutic approach on adipose tissue repair and regeneration.

miRNAs play an important role in the posttranscriptional regulation of target mRNA in a range of biological processes, including maintenance of stemness and modulation of mobilization, proliferation, and differentiation of ADSCs [2]. Recent studies demonstrated that some miRNAs played important regulatory roles in either promoting or inhibiting adipogenesis [3–5]. miR-150 has been extensively studied in B and T cells. Downregulation of miR-150 is critical to the formation of progenitor B cells and pre-B cells. Abnormal expression of miR-150 will affect the development of pre-B cells and progenitor B cells into mature B lymphocytes [6]. miR-150 can regulate embryonic development [7] and the development of B cells, T cells, and natural killer cells through targeting the transcription factor c-Myb [8, 9]. Recent studies have reported that miR-150 is also expressed in bone marrow mesenchymal stem cells (BMSCs). In myocardial ischemia, miR-150 promotes the mobilization and migration of BMSCs by acting on CXCR4 and then participates in the repair process of ischemic tissue [10]. In human embryonic stem cells, miR-150 promotes the differentiation of embryonic stem cells into endothelial cells by inhibiting the expression of target gene ZEB1 [11]. Recent study demonstrated that the level of miR-150 was upregulated in the subcutaneous adipose tissue of obese human subjects [12]. However, miR-150 expression in ADSCs and its role in the regulation of ADSC differentiation and proliferation have not been reported.

The Notch family members are highly conserved and have the effect of regulating the differentiation and proliferation of stem cells and progenitor cells [13]. Inhibition of Notch signaling can promote ADSCs to differentiate into a variety of cell types, including fat cells [14]. Notch3 belongs to the Notch family. It was previously reported that control of the Notch3 signal through miR-150 might have an important impact on T cell development [15]. It is not clear, whether miR-150 regulates the adipogenic differentiation of ADSCs by targeting Notch3 signaling.

In the present study, we investigated the mechanism of miR-150 in adipogenic differentiation of ADSCs. We demonstrated for the first time that miR-150 played an important role in ADSC differentiation towards the adipose by targeting Notch3.

## 2. Materials and Methods

### 2.1. Isolation and Culture of ADSCs

ADSCs were isolated from the inguinal white adipose tissue from miR-150 KO mouse in a C57BL/6 background and wild-type (WT) C57BL/6 mouse (4-6 weeks) according to a published protocol from our laboratory [16]. The ADSCs were cultured in DMEM/F12 (Catalogue Number: SH30023.01, Hyclone) containing 10% fetal bovine serum (FBS, Catalogue Number: S601P-500, SERA-PRO, South America) and 1% penicillin/streptomycin solution in a 5% CO_2_ humidified atmosphere at 37°C. The third-passage cells were identified with flow cytometry. Human ADSCs (hADSCs) were kindly provided by Stem Cell Bank, Chinese Academy of Sciences. The hADSCs were harvested from a female donor using lipoaspirate, and the cells were expanded for five passages prior to cryopreservation and shipment. hADSCs were cultured under the same conditions as the ADSCs from mouse.

### 2.2. Flow Cytometric Analysis

Third-passage mouse ADSCs were trypsinized, washed, and resuspended with PBS and incubated with antibody CD45 (Catalogue Number: 561087, BD Pharmingen, USA), CD44 (Catalogue Number: 561860, BD Pharmingen, USA), CD105 (Catalogue Number: 562759, BD Pharmingen, USA), or CD34 (Catalogue Number: 560238, BD Pharmingen, USA) conjugated with fluorescent dyes or isotype-matched IgG controls. Single-cell suspensions were incubated at 4°C for 30 min and then washed three times with PBS. After centrifugation, the cells were resuspended in 500 *μ*l PBS and assessed with fluorescence-activated cell sorting (FACS) analysis. Data were analyzed using the FlowJo software. Gating strategy was determined based on isotype control staining.

### 2.3. Cell Proliferation and Colony Forming Assay

A 3-(4,5-dimethylthiazol-2-yl)-2,5-diphenyl-tetrazolium bromide (MTT) assay was performed to evaluate cell proliferation rate at different time points. For the colony formation assay, ADSCs (1 × 10^4^) were seeded in a 10 cm dish and cultured for up to 14 days. Then, cells were washed with PBS, fixed with 4% paraformaldehyde in PBS, and stained with 0.5% crystal violet (Catalogue Number: 61135, Sigma-Aldrich) in methanol for 10 min at room temperature.

### 2.4. Induction of Differentiation

ADSCs were cultured in complete culture media in 6-well culture plates. Adipogenic differentiation was induced for 7 days in DMEM/F12 (Catalogue Number: SH30023.01, Hyclone) supplemented with 10% fetal bovine serum (FBS, Catalogue Number: S601P-500, SERA-PRO, South America), 1 *μ*m dexamethasone (Catalogue Number: D4902, Sigma-Aldrich), 10 *μ*m insulin (Catalogue Number: I8830, Solarbio, China), 200 *μ*m indomethacin (Catalogue Number: 1820, Sigma-Aldrich), and 0.5 mm 3-isobutyl-1-methylxanthine (Catalogue Number: 15879, Sigma-Aldrich). Oil red O staining as an indicator of intracellular lipid accumulation was performed, as described previously [17]. For quantitative assessment, the oil red O was dissolved in isopropanol (100%) and the absorbance was measured by a spectrophotometer at 510 nm. To confirm osteogenic differentiation, ADSCs were cultured with a specific induction medium consisting of 0.1 mM dexamethasone, 10 mM *β*-glycerophosphate (Catalogue Number: G9422, Sigma-Aldrich), and 50 *μ*M ascorbic acid (Catalogue Number: A4544, Sigma-Aldrich). To quantify ALP enzymatic activity, cells were cultured for 7 days, then washed three times with PBS, fixed with 4% paraformaldehyde for 2 minutes, and visualized by staining with an alkaline phosphatase detection kit (Catalogue Number: AP0100, Sigma-Aldrich).

### 2.5. Western Blot Analysis

The cells were washed twice with ice-cold PBS and lysed in RIPA buffer. The protein content of the lysate was determined using a BCA protein assay kit (Catalogue Number: P0012, Beyotime, Shanghai, China). The proteins were separated by 10% SDS polyacrylamide gels and transferred onto polyvinyl difluoride membranes. The membranes were blocked in 5% BSA in TBST for 1 hour at room temperature and then incubated with a primary antibody overnight at 4°C. The primary antibodies used were mouse anti-Oct-4 (Catalogue Number: A7920, 1 : 1000, ABclonal Technology), rabbit anti-Sox-2 (Catalogue Number: 27015, 1 : 1000, AbSci, USA), rabbit anti-Nanog (Catalogue Number: A3232, 1 : 1000, ABclonal Technology), anti-PPAR-*γ* (Catalogue Number: 2443, 1 : 1000, Cell Signaling, USA), rabbit anti-Notch3 (Catalogue Number: sc-515617, 1 : 1000, Santa Cruz, USA), rabbit anti-c/EBP*α* (Catalogue Number: 2295, 1 : 1000, Cell Signaling, USA), and mouse anti-*β*-actin (Catalogue Number: AB21800, 1 : 5000, AbSci, USA). The membranes were incubated with HRP-conjugated secondary anti-mouse or anti-rabbit antibodies and visualized via an enhanced chemiluminescence (ECL) kit (Catalogue Number: P0018S, Beyotime Biotechnology, Shanghai, China) using ChemiDoc XRS with Quantity One Software (Bio-Rad, Hercules, CA). Bands on immunoblots were analyzed by densitometry.

### 2.6. ADSC Transfection and Infection

The synthetic miR-150 mimics and negative controls were purchased from Invitrogen. Subconfluent ADSCs were washed with PBS and incubated in trypsin/EDTA, PBS, and collagenase. The cells were resuspended and pelleted at 800 g for 5 min and then resuspended in Opti-MEM® I Reduced Serum Medium (Catalogue Number: 31985070, Invitrogen). ADSCs cultured in six-well plates were transfected with miRNA mimics and siRNA using siPORT™ NeoFX™ Transfection Agent (Catalogue Number: AM-4511, Ambion, Applied Biosystems) according to the manufacturer's protocol.

A small hairpin (sh) RNA Notch3 expression vector (Plent-U6-GFP-Puro, 8.3 kb) or negative control was generated by company (Vigene, Shandong, China). The packaged lentiviruses used contained lentiviruses targeting Notch3 (shNotch3-1 and shNotch3-2) and the scrambled control (shNC). The shRNA target sequences were as follows: 5′-GCACCCAGTCCGCCCTGAGCAAATTCAAGAGATTTGCTCAGGGCGGACTGGGTGCTTTTT-3′ (shNC), 5′-GATCCGCGTGTGTAGACGGTGTCAATATTCAAGAGATATTGACACCGTCTACACACGTTTTTTA-3′ (Notch3-siR1), and 5′-GATCCGCTAGCTTCTCGTGTGCTTGTTTCAAGAGAACAAGCACACGAGAAGCTAGCTTTTTTA-3′ (Notch3-siR2).

For the overexpression of miR-150, recombinant adenovirus expressing miR-150 (adv-miR-150) and empty control adenovirus were constructed by Shandong Vigene Biosciences. The infection of adenovirus was performed in serum-free media. After 4 h, medium change was carried out with serum-containing fresh media. Cells were maintained at 37°C in a humidified atmosphere with 5% CO_2_ for 48 h and harvested for further studies. Under these circumstances, the transduction efficiency of the adenovirus reached almost 100%.

### 2.7. Dual Luciferase Reporter Assay

The synthetic fragment of Notch3 3′UTR containing the predicted seed match site with miR-150 or the mutant site was inserted between the SacI and XhoI cleavage sites of pmirGLO plasmid (Wuhan GeneCreate Biological Engineering Co., Ltd.), downstream of the renilla luciferase reporter gene. For luciferase report assay, the ADSCs were cotransfected with luciferase reporters, either wild-type or mutant Notch3 3′UTR, in combination with miR-150 mimics or negative control by using Lipofectamine 2000 (Catalogue Number: 11668019, Invitrogen). After 48 hours, the firefly and renilla luciferase activities were measured and analyzed according to the manufacturer's instruction (Catalogue Number: N1610, Promega). Renilla luciferase activity was normalized to firefly luciferase activity.

### 2.8. RNA Isolation and Quantitative PCR

Total RNA was extracted from ADSCs using Trizol (Catalogue Number: 15596018, Invitrogen, USA) according to the manufacturer's instructions. cDNA was synthesized using the RiboTM Reverse Transcription Kit (Catalogue Number: C10170, RiboBio, China). The reverse transcription reaction for miR-150 was carried out with Bulge-LoopTM miRNA qRT-PCR Primers (RiboBio, China). Relative expression was determined using U6 (RiboBio, China) as an internal control for miR-150. Quantitative real-time PCR was performed with three technical replicates on the ABI-7900HT Real-Time PCR System (Applied Biosystems) using the SYBR Green Master Mix (Catalogue Number: RR036A, Takara, Dalian, China).

### 2.9. Statistical Analysis

All the data were analyzed by one-way analysis of variance (ANOVA). The unpaired *t*-test was used for comparisons between two groups. *P* values < 0.05 were considered to be statistically significant.

## 3. Results

### 3.1. miR-150 Level Was Increased in ADSCs with Adipogenic Differentiation

ADSCs were isolated from the inguinal adipose tissue of C57 mice, cultured, and identified by flow cytometry using CD markers. The expression levels of blood cell-related marker CD45 and the hematopoietic stem cell marker CD34 were negative, while the mesenchymal stem cell-related markers CD44 and CD105 were highly expressed in isolated cells (Supplemental [Supplementary-material supplementary-material-1]). The adipogenic and osteogenic differentiation capacity of the ADSCs was also identified (Supplemental [Supplementary-material supplementary-material-1]). The results indicated that the isolated cells were ADSCs.

**Figure 1:**
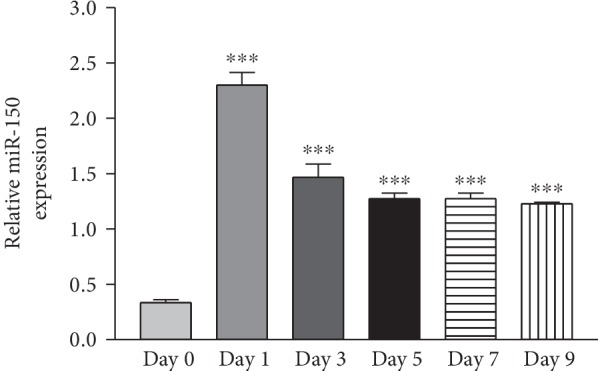
miR-150 was upregulated during adipogenic differentiation of ADSCs. Real-time PCR analysis of miR-150 expression in ADSCs with adipogenic induction at different time points. U6 was used as the internal control. ^∗∗∗^*P* < 0.0001 vs. day 0.

The ADSCs were induced by adipogenic differentiation for 1, 3, 5, 7, and 9 days, respectively. Real-time PCR analysis showed that miR-150 level was increased in the first day until the 9th day after adipogenic induction and peaked at 1 day after differentiation. This result suggested that miR-150 was involved in the adipogenic differentiation process in ADSCs (Figure 1).

### 3.2. miR-150 Deficiency Attenuated Adipogenic Differentiation in ADSCs

**Figure 2:**
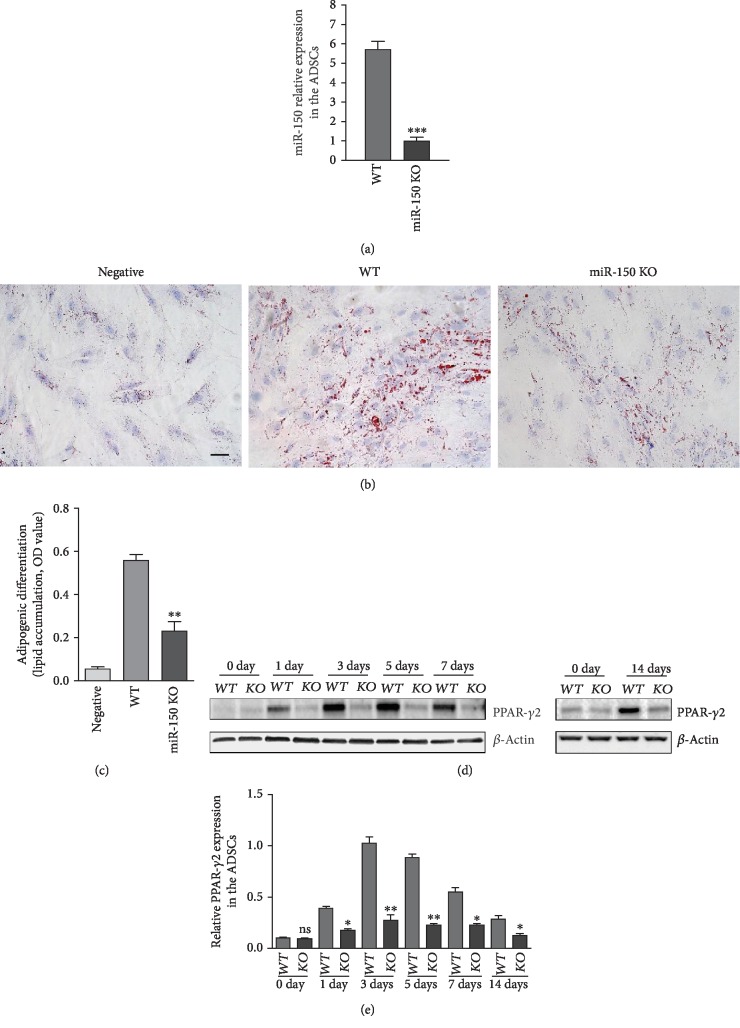
miR-150 deficiency attenuated adipogenic differentiation in ADSCs. (a) Real-time PCR analysis of miR-150 in ADSCs of WT and miR-150 KO mice. U6 was used as the internal control. ^∗∗∗^*P* < 0.0001 vs. WT mice. (b) Lipid formation assessed by oil red O staining after 7 days of adipogenic induction. Scale bar, 50 *μ*m. (c) Quantification of lipid accumulation (oil red O staining density). ^∗∗^*P* < 0.01 vs. the WT mice. (d) Western blot analysis of PPAR-*γ*2 expression in ADSCs. (e) Quantification of PPAR-*γ*2 expression in ADSCs. Results were normalized with *β*-actin. ^∗^*P* < 0.05 and ^∗∗^*P* < 0.01 vs. the WT mice. *n* = 3 independent experiments.

To assess the effect of miR-150 on adipogenesis, the ADSCs of WT mice and miR-150 KO mice were isolated. Real-time PCR was performed to detect the miR-150 expression in ADSCs. The expression of miR-150 was significantly lower in ADSCs of miR-150 KO mice compared with that of WT mice (Figure 2(a)). The result indicated efficient deletion of the miR-150 in ADSCs.

The ADSCs were incubated with adipogenic induction medium for 1, 3, 5, 7, and 14 days and stained by oil red O. The result showed that lipid accumulation was attenuated in ADSCs from miR-150 KO mice compared with WT mice (Figures 2(b) and 2(c)). The expression of PPAR-*γ*2, as the marker of adipocyte differentiation, in the ADSCs of miR-150 KO mice was also decreased compared with that of WT mice following adipogenic induction (Figures 2(d) and 2(e)). The result showed that miR-150 deficiency decreased adipogenic differentiation of ADSCs.

### 3.3. Overexpression of miR-150 Facilitated Adipogenic Differentiation in ADSCs

**Figure 3:**
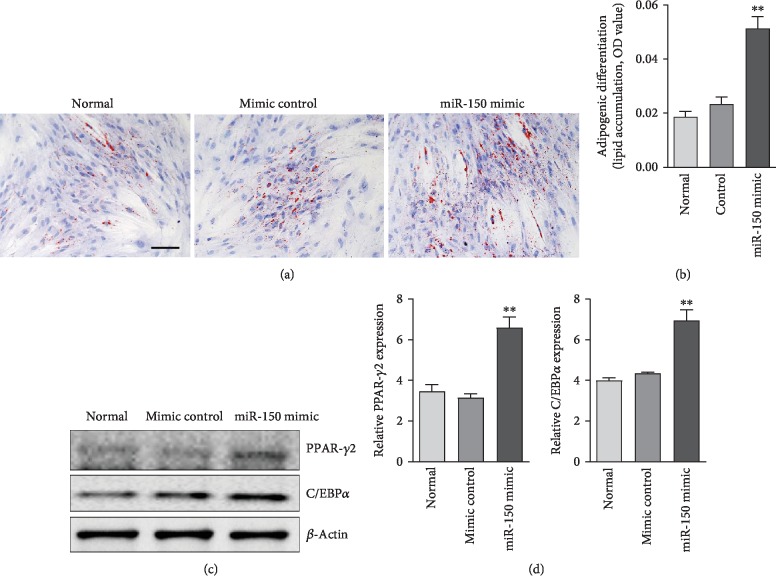
Overexpression of miR-150 increased the adipogenic differentiation in ADSCs. (a) Lipid formation assessed by oil red O staining after 7 days of adipogenic induction. Bar, 100 *μ*m. (b) Quantification of lipid accumulation (oil red O staining density). ^∗∗^*P* < 0.01 vs. the control group. (c) Western blot analysis of PPAR-*γ*2 and C/EBP*α* expression in ADSCs. (d) Quantification of PPAR-*γ*2 and C/EBP*α* expression in ADSCs. Results were standardized to *β*-actin. ^∗∗^*P* < 0.01 vs. the control group. *n* = 3 independent experiments.

To further explore the functional role of miR-150 on adipogenic differentiation in ADSCs, miRNA gain-of-function experiments were conducted using mmu-miR-150 mimic in ADSCs. Real-time PCR was performed to detect the miR-150 expression after overexpression by transfection method. The result indicated that miR-150 was increased in the ADSCs. The ADSCs were treated with adipogenic induction medium for 7 days and stained by oil red O. The result showed that miR-150 overexpression significantly increased the adipogenic differentiation of ADSCs. Western blot analysis revealed that overexpression of miR-150 increased expression of PPAR-*γ*2 and c/EBP*α* in ADSCs (Figure 3). These results demonstrate, for the first time, that miR-150 facilitates adipogenic differentiation in ADSCs.

### 3.4. Notch3 Was a Direct Downstream Target of miR-150

**Figure 4:**
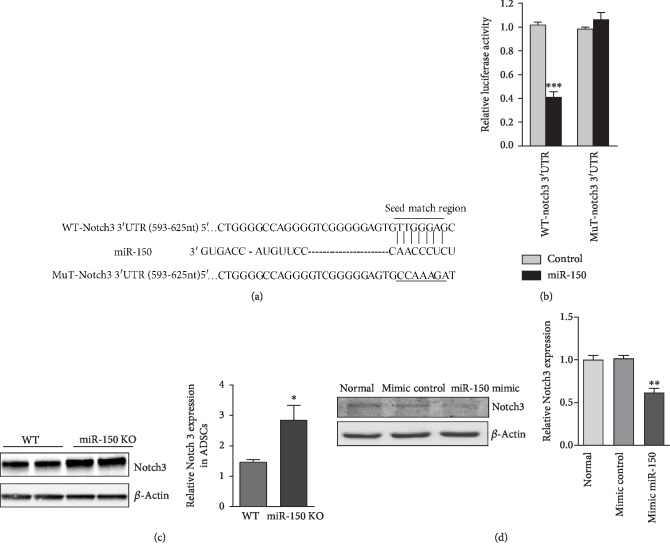
miR-150 inhibited Notch3 expression by targeting its 3′UTR. (a) Complementary of miR-150 seed sequence to the 3′UTR of Notch3. miR-150 complementary sequence within Notch3 3′UTR or its mutant sequence was inserted into the downstream of the luciferase construct. (b) Luciferase activity assay in ADSCs. Effects of miR-150 on luciferase activity of the reporter gene bearing wild-type or mutant 3′UTR of Notch3 in ADSCs. (c) Western blot analysis of Notch3 expression in ADSCs of WT and miR-150 KO mice. (d) Western blot analysis of Notch3 expression in ADSCs with miR-150 overexpression. Results were normalized with *β*-actin. ^∗^*P* < 0.05 and ^∗∗^*P* < 0.01 vs. the control group. *n* = 3 independent experiments.

To reveal the molecular mechanism underlying the regulation of ADSCs by miR-150, the potential downstream targets of miR-150 were searched in the TargetScan database. As shown in Figure 4(a), there was a potential binding site for miR-150 in the 3′UTR region of Notch3, which indicated that Notch3 might be targeted by miR-150. To investigate whether miR-150 inhibits the expression of Notch3 through the seed region in its 3′UTR, we inserted a 3′UTR sequence of Notch3 containing the target sequence for miR-150, or a fragment which target site was mutated, into a dual luciferase construct, pmirGLO, which was then cotransfected with miR-150 mimics to detect Notch3 protein translation via renilla luciferase activities. As shown in Figure 4(b), miR-150 repressed the luciferase activity through the 3′UTR of Notch3 compared with the miR-NC transfection control group. Additionally, miR-150 resulted in no effect on luciferase activity in the group inserted with mutated seed match site in 3′UTR or the control group without a 3′UTR sequence of Notch3.

To further confirm that the protein expression of Notch3 is regulated by miR-150, the Notch3 level was detected by loss- and gain-of-function analysis of miR-150 in ADSCs. The Western blot result showed that the level of Notch3 in the ADSCs of miR-150 KO mice was higher compared with that of WT mice. In addition, the expression of Notch3 was decreased in ADSCs with miR-150 overexpression (Figures 4(c) and 4(d)). The above data indicated that miR-150 could inhibit Notch3 expression.

### 3.5. Notch3 Inhibition Promoted the Adipogenic Differentiation in ADSCs

**Figure 5:**
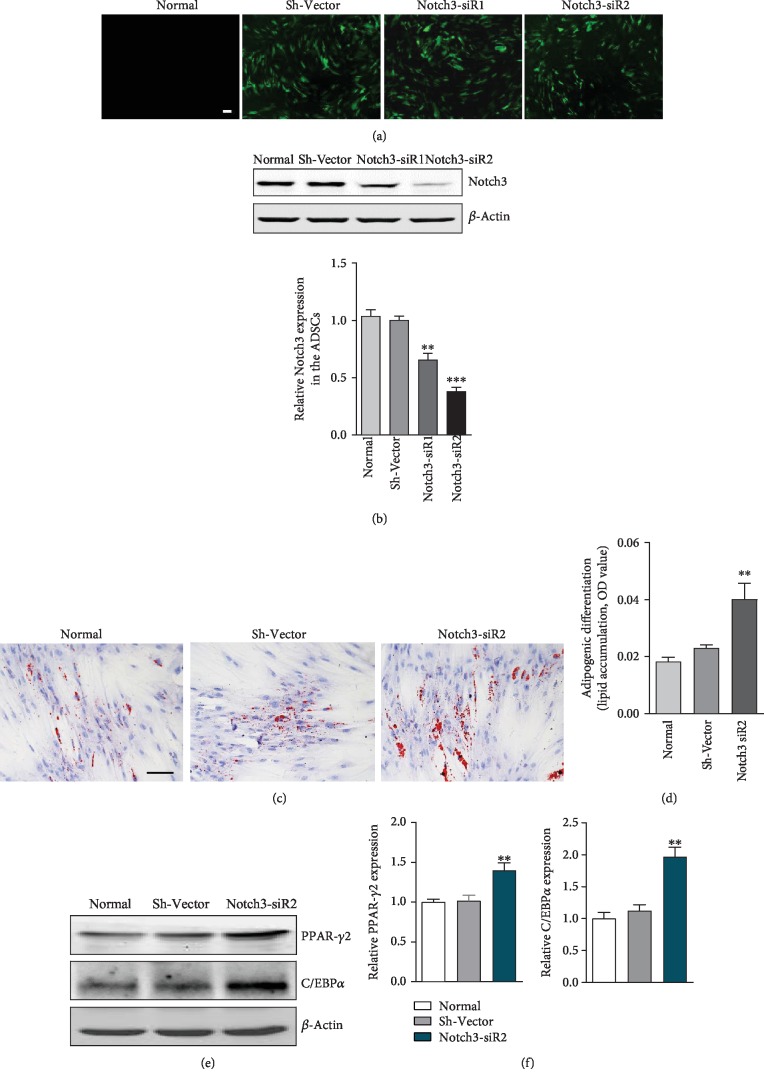
Notch3 depletion attenuated adipogenic differentiation in ADSCs. (a) ADSCs were infected with the lentiviruses and selected with puromycin for 1 week. The shRNA mediated knockdown of Notch3 in ADSCs. Bar, 200 *μ*m. (b) Western blot analysis of Notch3 expression in ADSCs. Results were normalized with *β*-actin. (c) Lipid formation assessed by oil red O staining after 7 days of adipogenic induction. Bar, 100 *μ*m. (d) Quantification of lipid accumulation (oil red O staining density). (e) Western blot analysis of PPAR-*γ*2 and C/EBP*α* expression in ADSCs. (f) Quantification of PPAR-*γ*2 and C/EBP*α* expression. Results were normalized with *β*-actin. ^∗∗^*P* < 0.01 and ^∗∗∗^*P* < 0.001 vs. the Sh-Vector group. *n* = 3 independent experiments.

We hypothesized that Notch3 has an antiadipogenic differentiation effect in ADSCs, and knockdown of Notch3 would enhance adipogenic differentiation in ADSCs. Two clones of sh-Notch3 knockdown were generated in ADSCs. As shown in Figure 5, Notch3 expression level was downregulated due to the transfection of Notch3 siRNAs, especially Notch3-siR2. Then, the Notch3-siR2 was used for further adipogenic differentiation experiment. After 7 days of incubation in adipogenic induction medium, oil red O staining and expression of adipo-specific markers (PPAR-*γ*2 and C/EBP*α*) showed that adipogenic differentiation of ADSCs was enhanced as a result of Notch3 inhibition (Figure 5).

### 3.6. miR-150 Increased Adipogenic Differentiation in hADSCs

**Figure 6:**
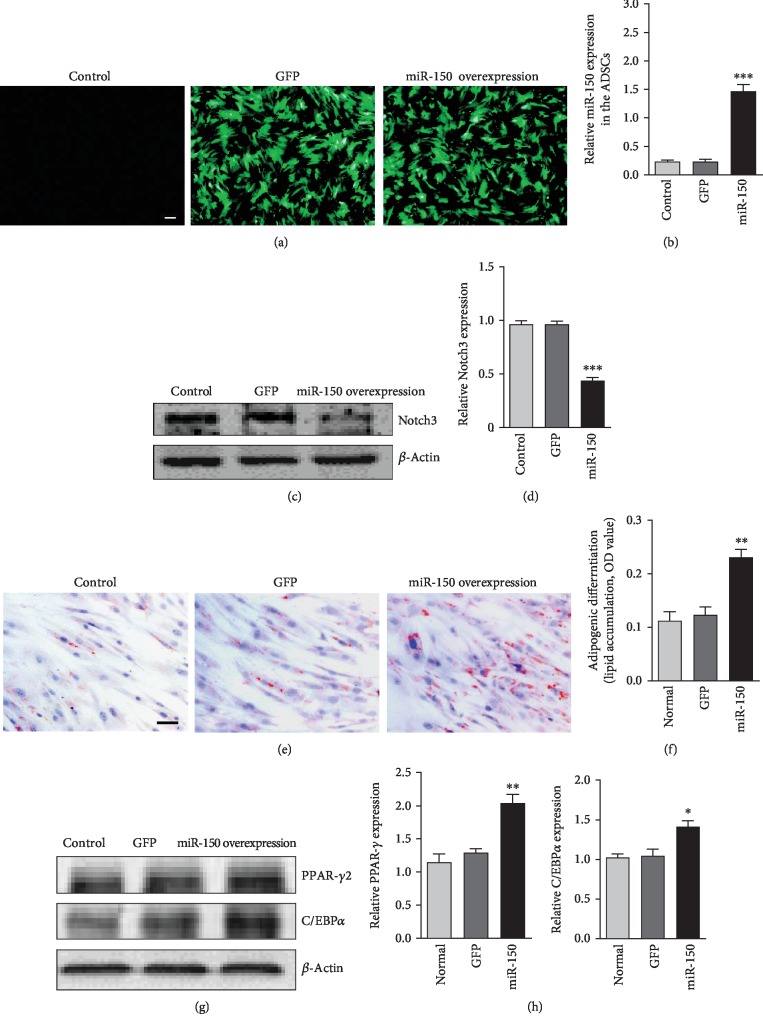
miR-150 increased adipogenic differentiation in hADSCs. (a) ADSCs were infected with the adv-miR-150 for 2 days. Bar, 200 *μ*m. (b) miR-150 levels were determined using real-time polymerase chain reaction (PCR). (c) Western blot analysis of Notch3 expression in ADSCs. (d) Results were normalized with *β*-actin. (e) Lipid formation assessed by oil red O staining after 7 days of adipogenic induction. Bar, 50 *μ*m. (f) Quantification of lipid accumulation (oil red O staining density). (g) Western blot analysis of PPAR-*γ* and C/EBP*α* expression in ADSCs. (h) Results were standardized to *β*-actin. ^∗^*P* < 0.05, ^∗∗^*P* < 0.01, ^∗∗∗^*P* < 0.001 vs. the GFP group. *n* = 3 independent experiments.

To further confirm the miR-150 effect on the adipogenesis in human stem cells, the effect of miR-150 on adipogenesis in hADSCs was also examined. The hADSCs (provided by Stem Cell Bank, Chinese Academy of Sciences) were infected with or without addition of adv-miR-150, then induced by adipogenic differentiation medium for 7 days. Western blot result showed that overexpression of miR-150 downregulated the expression of Notch3 in ADSCs. In addition, miR-150 overexpression significantly increased lipid accumulation in hADSCs and the expression of PPAR-*γ*2 and c/EBP*α* in ADSCs (Figure 3). The results demonstrated that miR-150 induced adipogenic differentiation in hADSCs and decreased Notch3 expression (Figure 6).

### 3.7. miR-150 Inhibited Proliferation of ADSCs

**Figure 7:**
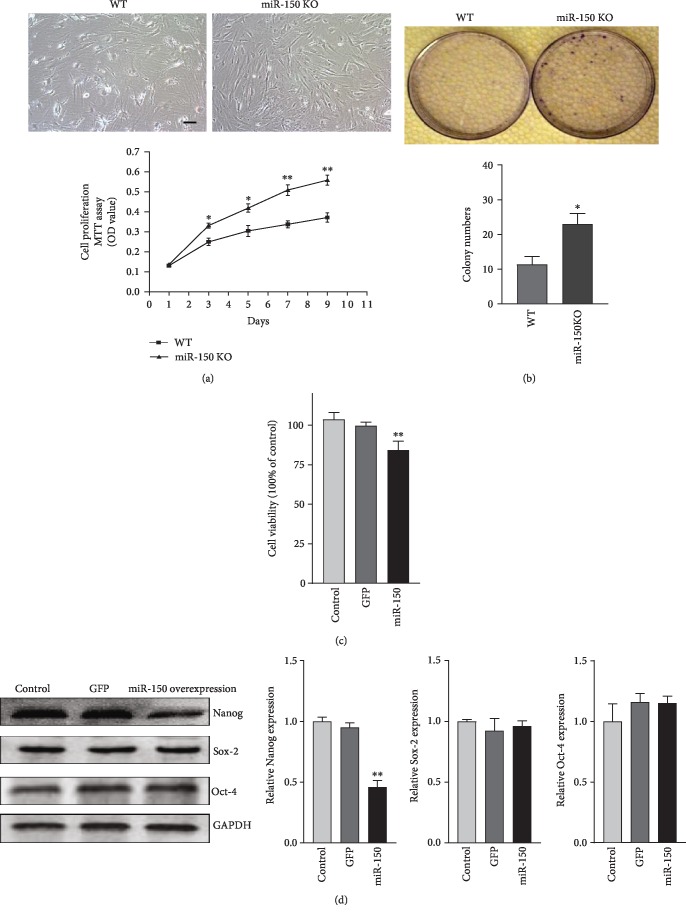
miR-150 inhibits cell proliferation in ADSCs. (a) MTT assay of the proliferation of ADSCs in WT and miR-150 KO mice over a period of 9 days. Bar, 100 *μ*m. (b) Colony formation of ADSCs cultured for 14 days. (c) MTT assay of the proliferation of ADSCs with miR-150 overexpression for 48 hours. (d) Western blot analysis of Nanog, Sox-2, and Oct-4 expression. Results were normalized with *β*-actin. ^∗^*P* < 0.05 and ^∗∗^*P* < 0.01 vs. the WT group. *n* = 3 independent experiments.

To investigate whether miR-150 regulates self-renewal in ADSCs, the primary WT and miR-150 KO ADSCs were cultured, and we performed the MTT assay and the clonogenic assay. The results showed that cell proliferation was increased in ADSCs of miR-150 KO mice compared with WT mice. At the same time, there were more colonies in ADSCs of miR-150 KO compared with WT mice (Figures 7(a)–7(c)).

However, with miR-150 overexpression, the cell proliferation of ADSCs was decreased. In addition, the expression of the pluripotency-associated transcription factor Nanog was significantly decreased in ADSCs with miR-150 overexpression, while Sox-2 and Oct-4 levels were unchanged (Figure 7(d)).

This finding indicated that miR-150 suppressed ADSCs' proliferation potential.

## 4. Discussion

miRNAs are a class of evolutionarily conserved endogenous noncoding single-stranded small RNA molecules. miRNAs are widely present in mammals and have a length of about 22 nt. The corresponding protein-coding genes are posttranscriptionally regulated by incomplete alignment with the 3′ untranslated region (3′UTR) sequence of the target mRNA. The most representative role of miRNAs is to induce specific targeted mRNA degradation or repression [6–8]. In some cases, the miRNA can upregulate the expression of the target gene by acting on the 5′ untranslated region or coding region of the target gene [9, 10]. Most miRNAs are tissue-specific and only play a role in specific tissues or cells [11]. Several miRNAs show the effect of promoting or inhibiting adipogenesis [12–14]. In this study, we found that miR-150 was expressed in mouse ADSCs. The miR-150 knockdown significantly decreased the capacity of adipogenic differentiation of ADSCs. As the abstract that we presented in the meeting [18], it is intriguing that overexpression of miR-150 significantly increased C/EBP*α* and PPAR-*γ* expression and lipid formation in ADSCs with adipogenic induction, indicating increased adipogenic differentiation. Furthermore, overexpression of miR-150 significantly decreased Notch3 expression in ADSCs. Moreover, miR-150 could affect ADSCs' proliferation. This study provided the first evidence that miR-150 regulated ADSCs' proliferation and differentiation.

Recent studies highlight that miR-150 is closely associated with lipid metabolism. In adipose tissue, miR-150 may regulate lipid metabolism and cytokine expression by targeting genes linked to lipid accumulation, such as adiponectin receptor 2 (AdipoR2) and peroxisome proliferator-activated receptor gamma coactivator-1*α* (PGC-1*α*) [19, 20]. In miR-150 knockout mice, miR-150 ablation lowers body weight, which is mediated by the effects on circulating leptin, food consumption, and lipid metabolism in adipocytes [21]. miR-150 expression was increased in the inguinal white adipose tissue of wild-type mice on a high-fat diet [20]. The similar result was also detected in subcutaneous adipose tissue of obese human [12]. However, the miR-150 effect on the adipogenic differentiation of ADSCs is not known. In this study, we reported that miR-150 was increased with adipogenic differentiation in ADSCs, suggesting that miR-150 was involved in adipogenesis. Forced overexpression of miR-150 promoted adipogenic differentiation in ADSCs, whereas inhibition of miR-150 prevented the commitment of adipocytes.

To determine how miR-150 acts as an adipogenic-promoting gene, we searched for physiological targets using bioinformatic analysis and found a 7 nt match site complementary to miR-150 in the 3′UTR of Notch3 mRNA. In the current study, dual luciferase reporter assay showed that miR-150 directly targeted Notch3 at the translational level. We found that overexpression of miR-150 and transduction of miR-150 inhibitors have opposing effects on Notch3 expression. The Notch signaling pathway consists of four transmembrane Notch receptors (Notch1-Noth4) and ligands (Delta-like1, Delta-like3, Delta-like4, Jagged1, and Jagged2) [22]. Osathanon et al. analyzed 14 percent of ADSCs from different people. They found that ADSCs with low adipogenic ability had higher expression levels of Notch2, Notch3, and Notch4 receptors and their ligands Jagged1 and Delta1 in ADSCs with high adipogenic ability compared with cell populations with high adipogenic ability. DAPT, as the notch signal inhibitor, can enhance ADSC adipogenic differentiation [14, 23]. Furthermore, overexpression of Notch intracellular segment, or Notch ligand Jagged, downregulates the expression of PPAR-*γ* and inhibits the accumulation of intracellular lipid [24]. Data from the above studies supported that Notch signaling inhibited the adipogenic differentiation of ADSCs. Our experiments demonstrated that Notch3 silence promoted adipogenic differentiation ability in ADSCs. The results were consistent with a recent experiment, which also indicated that Notch3 was involved in adipogenesis of hADSCs [25]. Previous study demonstrated that a *γ*-secretase inhibitor of Notch signaling could increase the adipogenesis of mouse ADSCs by promotion of PPAR-*γ* expression [26]. These findings warrant further mechanistic investigation into how Notch3 negatively regulates PPAR-*γ*.

Morphologically, ADSCs from WT mice displayed as larger, with an irregular and flat shape. However, ADSCs from miR-150 KO mice displayed as spindle shape and flat shape together. MTT and colony formation experiments showed that miR-150 KO could increase the cell proliferation. To further understand the mechanism, with miR-150 overexpression in ADSCs, the level of transcription factors Oct-4, Sox-2, and Nanog was investigated, which played a key role in stem cell self-renewal and pluripotency states [27]. Our result showed that miR-150 overexpression decreased the expression of Nanog and had no effect on the expression of Oct-4 and Sox-2 in ADSCs. Recently, one study showed that miR-150 inhibits proliferation of leukemia stem cells by targeting Nanog [28], which was similar to our results. The loss of Nanog also inhibited the proliferation of bone marrow stromal cells [29]. Furthermore, knockdown of Nanog in bone marrow stromal cells diminishes bone formation and promotes adipogenesis [29]. Nanog was shown to decrease in expression during adipogenesis much more significantly than that during osteogenesis [30].

## 5. Conclusions

In summary, this study provides the first evidence that miR-150 is expressed in ADSCs. The novel relationship between miR-150 and Notch3 is critical for adipogenesis. A new role for miR-150 in the promotion of adipogenic differentiation in ADSCs is mediated by inhibiting Notch3 signaling.

## Data Availability

The data used to support the findings of this study are available and included within the article and the supplementary information file.

## Supplementary Materials

Supplementary Materials Supplemental Figure 1: flow cytometry was used to characterize ADSCs (P3) isolated from C57 mice. Low expression of surface markers CD34 and CD45 and CD44 and CD105 was highly expressed in ADSCs. Supplemental Figure 2: ADSCs were investigated for their in vitro multilineage differentiation capacity. ADSCs were stained by oil red O at day 7 of adipogenic induction. ADSCs were stained by Alizarin Red S at day 21 of osteogenic induction. Bar, 20 *μ*m.

## References

[1] Volz A. C., Huber B., Kluger P. J. (2016). Adipose-derived stem cell differentiation as a basic tool for vascularized adipose tissue engineering. *Differentiation*.

[2] Chen J., Deng S., Zhang S. (2014). The role of miRNAs in the differentiation of adipose-derived stem cells. *Current Stem Cell Research & Therapy*.

[3] Kim Y. J., Hwang S. J., Bae Y. C., Jung J. S. (2009). MiR-21 regulates adipogenic differentiation through the modulation of TGF-beta signaling in mesenchymal stem cells derived from human adipose tissue. *Stem Cells*.

[4] Yang Z., Bian C., Zhou H. (2011). MicroRNA hsa-miR-138 inhibits adipogenic differentiation of human adipose tissue-derived mesenchymal stem cells through adenovirus EID-1. *Stem Cells and Development*.

[5] Price N. L., Fernandez-Hernando C. (2016). miRNA regulation of white and brown adipose tissue differentiation and function. *Biochim Biophys Acta*.

[6] Zhou B., Wang S., Mayr C., Bartel D. P., Lodish H. F. (2007). miR-150, a microRNA expressed in mature B and T cells, blocks early B cell development when expressed prematurely. *Proceedings of the National Academy of Sciences of the United States of America*.

[7] Lin Y. C., Kuo M. W., Yu J. (2008). c-Myb is an evolutionary conserved miR-150 target and miR-150/c-Myb interaction is important for embryonic development. *Molecular Biology and Evolution*.

[8] Xiao C., Calado D. P., Galler G. (2007). MiR-150 controls B cell differentiation by targeting the transcription factor c-Myb. *Cell*.

[9] Bezman N. A., Chakraborty T., Bender T., Lanier L. L. (2011). miR-150 regulates the development of NK and iNKT cells. *The Journal of Experimental Medicine*.

[10] Tano N., Kim H. W., Ashraf M. (2011). microRNA-150 regulates mobilization and migration of bone marrow-derived mononuclear cells by targeting Cxcr4. *PLoS One*.

[11] Luo Z., Wen G., Wang G. (2013). MicroRNA-200C and -150 play an important role in endothelial cell differentiation and vasculogenesis by targeting transcription repressor ZEB1. *Stem Cells*.

[12] Martinelli R., Nardelli C., Pilone V. (2010). miR-519d overexpression is associated with human obesity. *Obesity (Silver Spring)*.

[13] Kadesch T. (2004). Notch signaling: the demise of elegant simplicity. *Current Opinion in Genetics & Development*.

[14] Osathanon T., Subbalekha K., Sastravaha P., Pavasant P. (2012). Notch signalling inhibits the adipogenic differentiation of single-cell-derived mesenchymal stem cell clones isolated from human adipose tissue. *Cell Biology International*.

[15] Ghisi M., Corradin A., Basso K. (2011). Modulation of microRNA expression in human T-cell development: targeting of NOTCH3 by miR-150. *Blood*.

[16] Tian X., Fan J., Yu M. (2014). Adipose stem cells promote smooth muscle cells to secrete elastin in rat abdominal aortic aneurysm. *PLoS One*.

[17] Wang J. Q., Fan J., Gao J. H., Zhang C., Bai S. L. (2013). Comparison of in vivo adipogenic capabilities of two different extracellular matrix microparticle scaffolds. *Plastic and Reconstructive Surgery*.

[18] Fan J., Li X., Kong D. Y. (2017). MiR-150 induces adipogenic differentiation of adipose-derived stem cells by regulating notch3. *Annal of Anatomy*.

[19] Garvey W. T. (2014). MicroRNA-150 regulates lipid metabolism and inflammatory response. *Journal of Metabolic Syndrome*.

[20] Chou C. F., Lin Y. Y., Wang H. K. (2014). KSRP ablation enhances brown fat gene program in white adipose tissue through reduced miR-150 expression. *Diabetes*.

[21] Kang M., Liu X., Fu Y., Timothy Garvey W. (2018). Improved systemic metabolism and adipocyte biology in miR-150 knockout mice. *Metabolism*.

[22] Shan T., Liu J., Wu W., Xu Z., Wang Y. (2017). Roles of notch signaling in adipocyte progenitor cells and mature adipocytes. *Journal of Cellular Physiology*.

[23] Song B. Q., Chi Y., Li X. (2015). Inhibition of notch signaling promotes the adipogenic differentiation of mesenchymal stem cells through autophagy activation and PTEN-PI3K/AKT/mTOR pathway. *Cellular Physiology and Biochemistry*.

[24] Ugarte F., Ryser M., Thieme S. (2009). Notch signaling enhances osteogenic differentiation while inhibiting adipogenesis in primary human bone marrow stromal cells. *Experimental Hematology*.

[25] Sandel D. A., Liu M., Ogbonnaya N., Newman J. J. (2018). Notch3 is involved in adipogenesis of human adipose-derived stromal/stem cells. *Biochimie*.

[26] Huang Y., Yang X., Wu Y. (2010). Gamma-secretase inhibitor induces adipogenesis of adipose-derived stem cells by regulation of Notch and PPAR-gamma. *Cell Proliferation*.

[27] Kallas A., Pook M., Trei A., Maimets T. (2014). Assessment of the potential of CDK2 inhibitor NU6140 to influence the expression of pluripotency markers NANOG, OCT4, and SOX2 in 2102Ep and H9 cells. *International Journal of Cell Biology*.

[28] Xu D. D., Zhou P. J., Wang Y. (2016). miR-150 suppresses the proliferation and tumorigenicity of leukemia stem cells by targeting the Nanog signaling pathway. *Frontiers in Pharmacology*.

[29] Bais M. V., Shabin Z. M., Young M., Einhorn T. A., Kotton D. N., Gerstnefeld L. C. (2012). Role of Nanog in the maintenance of marrow stromal stem cells during post natal bone regeneration. *Biochemical and Biophysical Research Communications*.

[30] Katsara O., Mahaira L. G., Iliopoulou E. G. (2011). Effects of donor age, gender, and in vitro cellular aging on the phenotypic, functional, and molecular characteristics of mouse bone marrow-derived mesenchymal stem cells. *Stem Cells and Development*.

