# Supplementary material for: MicroRNA-150 Modulates Adipogenic Differentiation of Adipose-Derived Stem Cells by Targeting Notch3
Source: Stem Cells Int. 2019 Oct 30;2019:2743047. doi: 10.1155/2019/2743047 (PMC6875317; doi:10.1155/2019/2743047)
Supplement: Supplementary Materials — Supplemental Figure 1: flow cytometry was used to characterize ADSCs (P3) isolated from C57 mice. Low expression of surface markers CD34 and CD45 and CD44 and CD105 was highly expressed in ADSCs. Supplemental Figure 2: ADSCs were investigated for their in vitro multilineage differentiation capacity. ADSCs were stained by oil red O at day 7 of adipogenic induction. ADSCs were stained by Alizarin Red S at day 21 of osteogenic induction. Bar, 20 μm. [file 2743047.f1.doc]

**Supplementary figures**

**
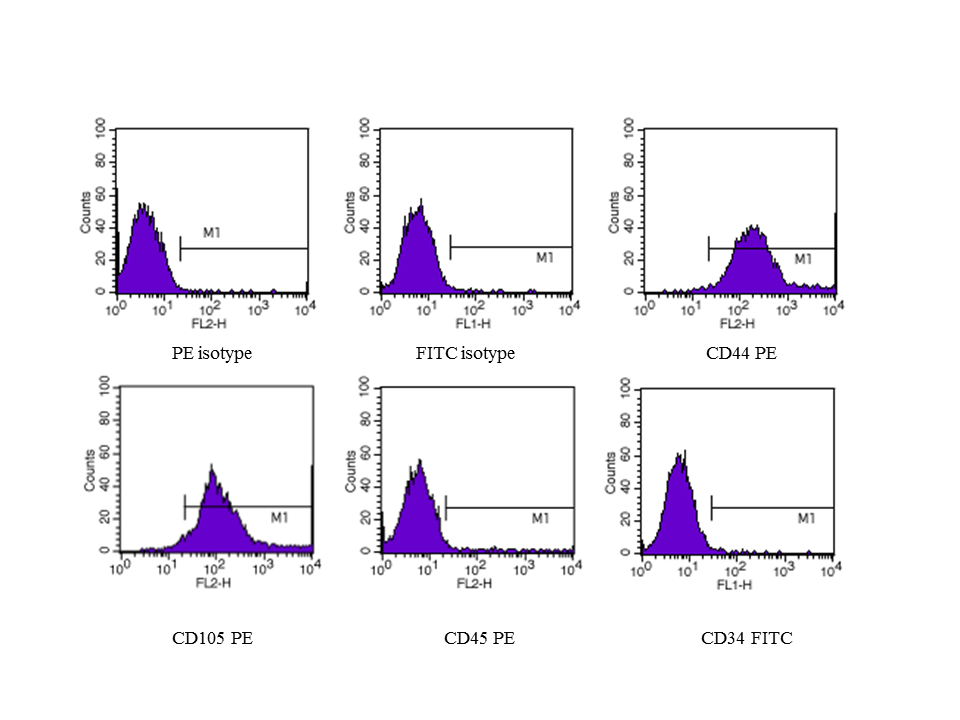
**

**Supplemental Figure 1.** Flow cytometry was used to characterize ADSCs (P3) isolated from C57 mice. Low expression of surface markers CD34 and CD45, and CD44 and CD105 were highly expressed in ADSCs.


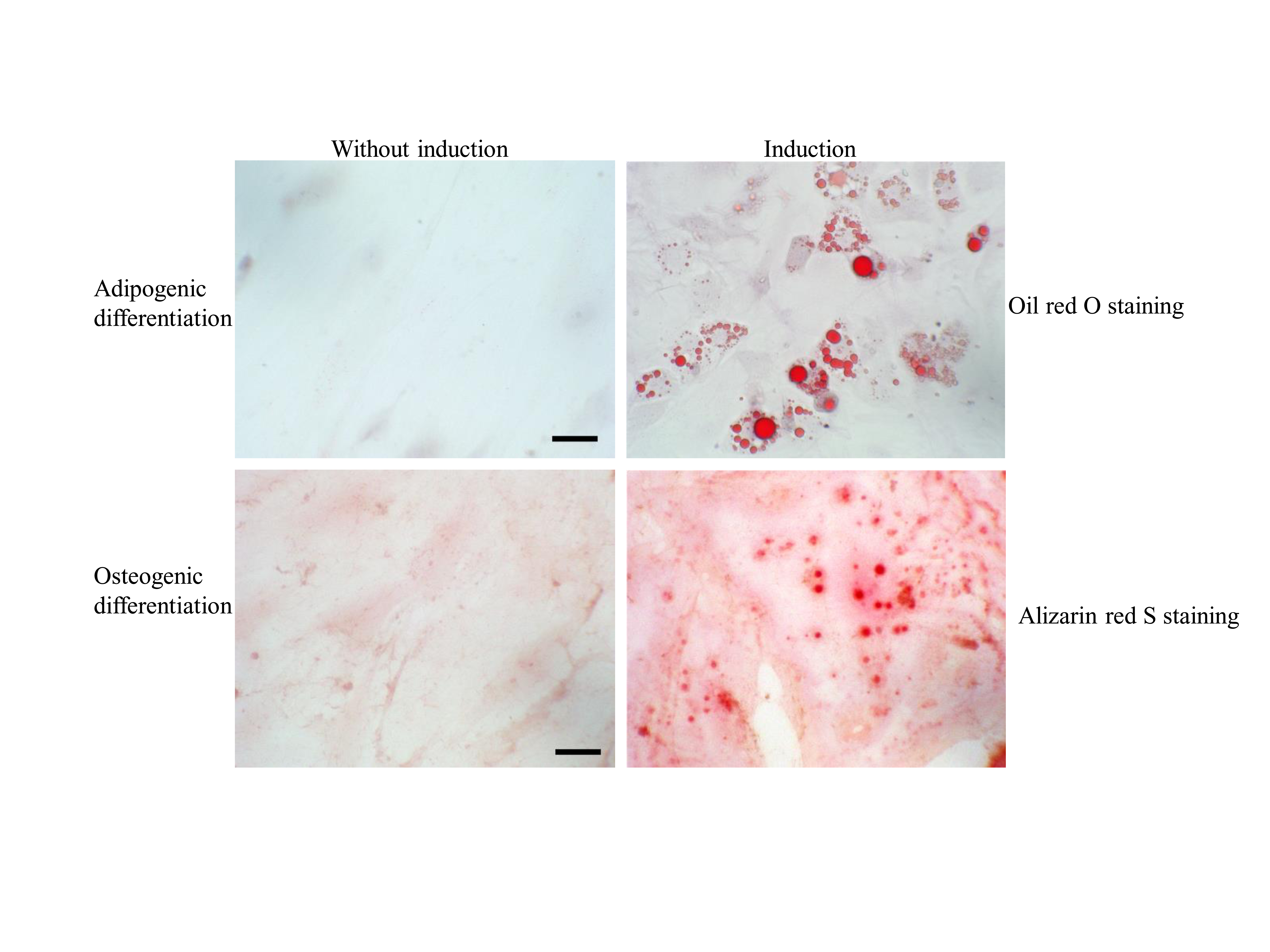


**Supplemental Figure 2.** ADSCs were investigated for their in vitro multilineage differentiation capacity. ADSCs were stained by oil Red O at day 7 of adipogenic induction. ADSCs were stained by Alizarin Red S at day 21 of osteogenic induction. Bar, 20μm.
